# Androgen receptor status is highly conserved during tumor progression of breast cancer

**DOI:** 10.1186/s12885-015-1897-2

**Published:** 2015-11-09

**Authors:** André Grogg, Mafalda Trippel, Katrin Pfaltz, Claudia Lädrach, Raoul A. Droeser, Nikola Cihoric, Bodour Salhia, Martin Zweifel, Coya Tapia

**Affiliations:** 1 Division of Clinical Pathology, Institute of Pathology, University of Bern, Bern, Switzerland; 2 Department of Surgery, University Hospital Basel, Basel, Switzerland; 3 Department of Radiation Oncology, Bern University Hospital, and University of Bern, Freiburgstrasse, 3010 Bern, Switzerland; 4 Translational Genomics Research Institute, Phoenix, USA; 5 Department of Medical Oncology, Bern University Hospital, Bern, Switzerland; 6 University Cancer Center, Breast Center, Inselspital Bern, Bern, Switzerland; 7 Department of Translational Molecular Pathology, University of Texas MD Anderson Cancer Center Life Science Plaza, 2130 W. Holcombe, Blvd. Unit 2951, Houston, TX 77030 USA

**Keywords:** Androgen receptor, Breast cancer, Metastasis, Recurrence

## Abstract

**Background:**

With the advent of new and more efficient anti-androgen drugs targeting androgen receptor (AR) in breast cancer (BC) is becoming an increasingly important area of investigation. This would potentially be most useful in triple negative BC (TNBC), where better therapies are still needed. The assessment of AR status is generally performed on the primary tumor even if the tumor has already metastasized. Very little is known regarding discrepancies of AR status during tumor progression. To determine the prevalence of AR positivity, with emphasis on TNBCs, and to investigate AR status during tumor progression, we evaluated a large series of primary BCs and matching metastases and recurrences.

**Methods:**

AR status was performed on 356 primary BCs, 135 matching metastases, and 12 recurrences using a next-generation Tissue Microarray (ngTMA). A commercially available AR antibody was used to determine AR-status by immunohistochemistry. AR positivity was defined as any nuclear staining in tumor cells ≥1 %. AR expression was correlated with pathological tumor features of the primary tumor. Additionally, the concordance rate of AR expression between the different tumor sites was determined.

**Results:**

AR status was positive in: 87 % (307/353) of primary tumors, 86.1 % (105/122) of metastases, and in 66.7 % (8/12) of recurrences. TNBC tested positive in 11.4 %, (4/35) of BCs. A discrepant result was seen in 4.3 % (5/117) of primary BC and matching lymph node (LN) metastases. Three AR negative primary BCs were positive in the matching LN metastasis, representing 17.6 % of all negative BCs with lymph node metastases (3/17). Two AR positive primary BCs were negative in the matching LN metastasis, representing 2.0 % of all AR positive BCs with LN metastases (2/100). No discrepancies were seen between primary BC and distant metastases or recurrence (n = 17).

**Conclusions:**

Most primary (87 %) and metastasized (86.1 %) BCs are AR positive including a significant fraction of TNBCs (11.4 %). Further, AR status is highly conserved during tumor progression and a change only occurs in a small fraction (4.1 %). Our study supports the notion that targeting AR could be effective for many BC patients and that re-testing of AR status in formerly negative or mixed type BC’s is recommended.

## Background

The androgen receptor (AR) is located on the long arm of the X chromosome (Xq12) and acts, upon ligand binding, as a transcription factor [1]. AR signaling has well documented roles in embryogenesis of both genders and plays an important role in mammary gland development in females [2]. The role of AR signaling in breast carcinogenesis is seemingly complex and is currently an area of intense investigation. AR signaling may have a dual role of both inhibiting and promoting cell proliferation. Inhibition of proliferation and cell growth was seen in hormone receptor positive and triple negative BC (TNBC) cell lines [3–5], respectively. Tumor growth due to AR activity was also shown for apocrine BCs [6]. In a Phase II clinical trial it was shown that patients with AR positive BC had a benefit from anti-androgenic therapy [7] indicating that targeting AR might be a therapeutic option. These results led to many subsequent clinical trials investigating anti-androgen therapy in BCs patients, especially in patients where no other targeted therapies were available, as in triple negative BC, and in patients with advanced disease [8] (ClinicalTrials.gov). Therefore, it is of great clinical value to know the prevalence of AR positivity in BC at all stages and molecular subtypes. Since receptor conversion between primary and metastatic sites has been observed for estrogen (ER), progesterone (PgR) and Her2 receptor [9, 10], we also assessed how AR expression may change with metastasis and recurrence. AR changes during tumor progression would have important clinical implications for patient selection of anti-AR therapy.

## Methods

### Patients

The patient cohort described in this study has been previously reported [11]. Briefly, patients diagnosed with therapy naïve, unilateral minimum pT1b primary BC diagnosed between 2005 and 2011 at the Institute of Pathology, University of Bern, Switzerland were included. Next generation tissue microarrays (ngTMAs) were constructed from the primary BCs, matched distant or lymph node metastases, and local and distant recurrences. Recurrence was defined as tumor manifestation >3 months (median: 24 months; range: 8–82 months) after initial surgery of the primary tumor. From 135 patients matching metastases, of these included, 129 axillary lymph nodes and 6 distant metastases were available. From 12 patients recurrences were available included 10 local-regional recurrences and 2 distant recurrences. The median age at diagnosis was 67 years (range: 31–98). The study was approved by the ethical committee of the University of Bern (Registration: 200/2014). The approval of the ethical committee includes a waiver of consent for retrospective TMA based studies based on material archived at the Institute of Pathology, University of Bern, Switzerland. Patient characteristics are recorded in Table 1.

**Table 1:** Patients characteristics (*n* = 356)

Features	*n* (%)
Age at diagnosis	median 67 yrs (range:31–98)
Histological subtypes	
No special type (NST)	253 (71.1)
Lobular	51 (14.3)
Mucinous	13 (3.7)
Carcinoma with medullary-like features	11 (3.1)
Ductulo-lobular	6 (1.7)
Tubular	4 (1.1)
Micropapillary	3 (0.8)
Cribriform	2 (0.6)
Glycogen rich	1 (0.3)
Mixed	12 (3.3)
Grading (Nottingham)	
G1	41 (11.5)
G2	194 (54.5)
G3	121 (34.0)
T-category (UICC 7th edition)	
T1	150 (42.1)
T2	167 (46.9)
T3	21 (5.9)
T4	18 (5.1)
N-category (UICC 7th edition)	
N0	146 (41.0)
N1mi	18 (5.1)
N1	102 (28.6)
N2	27 (7.6)
N3	22 (6.2)
no lymph nodes	41 (11.5)
Estrogen receptor	
positive (≥1 %)	308 (86.5)
negative (<1 %)	48 (13.5)
Progesterone receptor	
positive (≥1 %)	258 (72.5)
negative (<1 %)	98 (27.5)
Her2 Status	
positive	37 (10.4)
negative	316 (88.7)
Equivocal	1 (0.3)
no data	2 (0.6)
Proliferation fraction (MIB-1)	
High (≥20 %)	57 (16.0)
low (<20 %)	297 (83.4)
no data	2 (0.6)
Molecular subtypes (St. Gallen 2013)	
Luminal A	197 (55.4)
Luminal B (Her2 negative)	82 (23.1)
Luminal B (Her2 positive)	24 (6.7)
Her2	10 (2.8)
Triple negative	35 (9.8)
no data	8 (2.2)

### Next-generation Tissue Microarray (ngTMA)

Prior to ngTMA construction all primary BCs, positive lymph nodes, distant metastasis, and recurrences underwent pathological review (CT, MT, KP) for diagnostic confirmation. The primary tumors were classified according to the WHO classification 2013 [12]. ngTMA construction was performed as previously described [11, 13]. In brief, for TMA construction the most suitable, as per the discretion of the pathologists, formalin-fixed paraffin-embedded (FFPE) tissue block, was selected for each tumor sample. Corresponding H&E slides were scanned and up-loaded on the digital platform. The annotations were made on the scanned slides and afterwards the automated arrayer precisely punched the annotated areas out of the donor block into a new recipient block (ngTMA). Multiple punches from primary tumors, lymph nodes and recurrences were taken for ngTMA construction. In 97.8 % the primary tumors were represented by 6 punches. From the metastasis and recurrence, 2 punches (duplicates) were included in 82.2 % of the cases.

### Immunohistochemistry of androgen receptor

For the assessment of AR status a monoclonal anti-human androgen receptor antibody (clone AR441, Dako, Glostrup, Denmark) was used (1:100 dilution). A positive AR status was defined as average of ≥1 % positive tumor nuclei regardless of staining intensity as previously described [14]. The AR status of each tumor and corresponding metastasis or recurrence samples was evaluated without knowledge of sample annotation. The scoring was conducted according to the REMARK Guidelines for biomarkers [15].

Results on ER, PgR, Her2, and MIB-1 were available from our previous study [11], and the tumors were classified into the molecular subtypes according to the St. Gallen 2013 criteria [16].

### Statistics

The Chi-Square test was used to calculate significant differences between categorical variables. A *p*-value <0.05 was considered statistically significant. Analyses were carried out using SPSS 21 (IBM, Armonk, USA).

## Results

AR status was informative in 99.2 % (353/356) of primary BCs, 90.4 % (122/135) of metastases (lymph nodes: 117/129; distant metastasis: 5/6) and in 100 % (12/12) of the recurrences (loco-regional: 10/12; distant: 2/12). The majority of BCs were AR positive (≥75 %) with the exception of BCs with medullary-like features (36.4 %).

### AR status in primary BC and correlation with pathological parameters

Primary BCs were AR positive in 87 % (307/353) and showed a significant (p < 0.001) correlation with a positive ER (96.7 %; 295/305) and PgR (96.9 %; 247/255) status, and a low proliferation index of <20 % (93.2 %; 275/295).

ER negative primary BCs showed a positive AR status in 25 % (12/48), PgR negative BCs in 61.2 % (60/98), while ER and PgR negative BCs had a positive AR status in 26.7 % (12/45). A negative AR status correlated significantly (*p* < 0.001) with high tumor grade (G3) (37/120).

Luminal A were AR positive in 98.5 % (192/195), luminal B (Her2 negative) in 91.4 % (74/81), and all luminal B (Her2 positive) were AR positive (24/24). The Her2 and TNBC subtype were AR positive in 80 % (8/10) and in 11.4 % (4/35), respectively.

### AR status in metastases and recurrence

Lymph node metastases were AR positive in 85.5 % (100/117), distant metastases in 100 % (5/5) and recurrences in 66.7 % (8/12). The mean percentage of AR positive cells among recurrent BCs was: 43.1 % in primary tumors and 39.9 % in recurrences. AR negative BCs stayed negative in primary tumors and recurrences (mean: 0 % AR staining each). The results are summarized in Table 2.

**Table 2:** Primary breast cancer and androgen receptor status

Features	AR status *n* (%)		
	Positive (≥1 %)	Negative (<1 %)	*p*-value
Primary BC	307 (87.0)	46 (13.0)	
Metastases	105 (86.1)	17 (13.9)	
Recurrences	8 (66.7)	4 (33.3)	
Histological subtypes			
No special type (NST)	218 (86.5)	34 (13.5)	
Lobular	49 (98.0)	1 (2.0)	
Mucinous	10 (83.3)	2 (16.7)	
Carcinoma with medullary-like features	4 (36.4)	7 (63.6)	
Ductulo-lobular	6 (100)	0 (0)	
Tubular	3 (75)	1 (25)	
Micropapillary	3 (100)	0 (0)	
Cribriform	2 (100)	0 (0)	
Glycogen rich	1 (100)	0 (0)	
Mixed	12 (100)	0 (0)	
Grading (Nottingham)			
G1	38 (92.7)	3 (7.3)	
G2	186 (96.9)	6 (3.1)	
G3	83 (69.2)	37 (30.8)	<0.001
T-category (UICC 7th edition)			
1	138 (93.2)	10 (6.8)	
2	137 (82.5)	29 (17.5)	0.017
3	16 (76.2)	5 (23.8)	
4	16 (88.9)	2 (11.1)	
N-category (UICC 7th edition)			
N0	123 (85.4)	21 (14.6)	0.595
N1mi	18 (100.0)	0 (0.0)	
N1	87 (86.1)	14 (13.9)	
N2	21 (77.8)	6 (22.2)	
N3	21 (95.5)	1 (0.5)	
Estrogen receptor			
Positive (≥1 %)	295 (96.7)	10 (3.3)	<0.0001
Negative (<1 %)	12 (25)	36 (75)	
Progesterone receptor			
Positive (≥1 %)	247 (96.9)	8 (3.1)	<0.0001
Negative (<1 %)	60 (61.2)	38 (38.8)	
Her2 Status			
Positive	34 (91.9)	3 (8.1)	0.585
Negative	270 (86.3)	43 (13.7)	
Equivocal	1 (100)	0 (0)	
Proliferation fraction (MIB-1)			
High (≥20 %)	31 (55.4)	25 (44.6)	<0.0001
Low (<20 %)	275 (93.2)	20 (6.8)	
Molecular subtypes (St. Gallen 2013)			
Luminal A	192 (98.5)	3 (1.5)	<0.0001
Luminal B (Her2 negative)	74 (91.4)	7 (8.6)	
Luminal B (Her2 positive)	24 (100.0)	0 (0.0)	
Her2	8 (80)	2 (20)	
Triple negative	4 (11.4)	31 (88.6)	

*AR* Androgen receptor, *BC* Breast cancer

### Discordant AR status

A discordant AR status between primary BC and matched metastatic samples was observed in 4.1 % (5/122) of cases tested. However, a discrepant AR status was only seen between primary BC and matched lymph node metastases (4.3 %; 5/117), but not between primary BC and distant metastasis. Two AR positive primary BCs had a negative corresponding lymph node metastasis (2.0 %; 2 of 100 AR positive primary BC with evaluable matching lymph nodes). Three negative primary BCs had an AR positive lymph node metastasis (17.6 %; 3 of 17 AR negative primary BCs with evaluable matching lymph nodes). No discordant AR status was observed between the primary BC and distant metastases or recurrences.

### Re-evaluation of discordant AR status

**Fig. 1:**
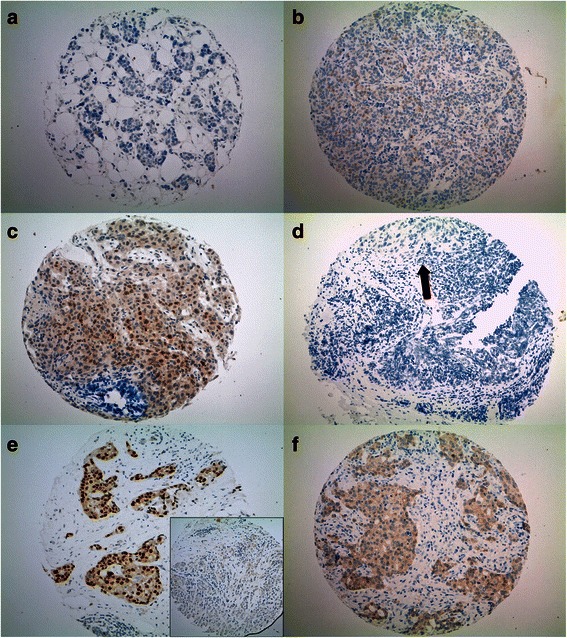
Examples of discrepant cases. **a**-**f**: TMA punches of matched primary BCs and matched lymph node metastases stained for the androgen receptor (AR) (100x magnification). **a**-**b**: patient ID 348; **c**-**d**: patient ID 47, **e**-**f**: patient ID 356. **a** Primary no special type (NST) BC with a negative androgen receptor (AR) status (<1 % positive tumor cells) and matched lymph node metastasis (**b**) with a positive, nuclear brown AR staining (1 % positive tumor cells). **c** Positive primary NST BC (5 % positive tumor cells) with some cytoplasmatic background and matched lymph node metastasis (**d**) with a negative AR status. The black arrow is pointing to the metastatic cells. On the bottom of the arrow some crush artifacts are visible. **e** Ductulo-lobular, primary BC with a positive and a negative (inlet) tumor component for AR. The matched lymph node metastasis (**f**) shows a negative AR status and some cytoplasmatic background

To confirm the discordant results, we re-evaluated the 5 discordant primary BC and their matching lymph node metastases. In two discordant cases (ID 237, 248), the primary BC was negative and their matched metastases were positive, but the AR status was close to the cut-off of ≥1 % for each. In another case (ID 204), the primary was negative but the lymph node metastasis was positive. In this case, the final score was not close to the cut-off of ≥1 %. In the fourth BC (ID 47), the primary BC was positive but the lymph node tissue was negative. The lymph node metastasis in this case was frozen prior to formalin-fixation (sentinel) and showed some crush artifacts, which could explain the negative score. The fifth case (ID 356) was of the ductulo-lobular histological subtype. The primary BC showed punches with both, a negative and a positive AR status. The primary tumor was signed off as AR positive but the metastasis was negative. The results are summarized in Table 3 and examples of discordance are given in Fig. 1.

**Table 3:** Discrepant AR status of primary BC and matched lymph node metastasis

	Androgen receptor status		
Patient ID	Primary BC	Lymph node metastasis	Comments
204	negative	positive	Fixation? tumor heterogeneity?
237	negative	positive	Evaluation (staining close to the cut-off of ≥1 %)?
348	negative	positive	Evaluation (staining close to the cut-off of ≥1 %)?
47	positive	negative	Pre-analytic handling? fixation? tumor heterogeneity?
356	positive	negative	Tumor heterogeneity

*BC* Breast cancer

## Discussion

For decades, the steroid hormone receptors ER and PgR have been well known therapeutic targets in BC [17] but considering AR therapy lagged in BC. In part, this lagging could be attributed to failure of early attempts of targeting AR to show benefit in BC [18]. Nevertheless, targeting AR is now becoming increasingly researched especially in patients with few targeted therapeutic options such as patients with TNBCs. Certainly, based on the results of our study and others [5] one could argue that anti-androgenic therapy should also be considered in AR positive BCs with ER and PgR positive, advanced, and recurrent disease, and/or in tumors which have become resistant to previous anti-estrogen therapy.

From a biological and therapeutic point of view, the dynamics of AR status during tumor progression is an important consideration. In a recent study, which included a small series of TNBC with matched recurrences (*n* = 16) and lymph node metastases (*n* = 46), it was shown that AR discrepancies between primary tumors and metastasis did not occur [19]. Our study confirmed, that AR status is highly preserved during tumor progression, but we did identify a few samples (*n* = 5) with discordant AR status from the primary tumor to the lymph node metastases. Due to the small number of distant metastases (*n* = 5) and recurrences (*n* = 12) included in our study and in the study by McNamara et al. [19], the degree to which AR discrepancies occurs in these cases may not have been adequately captured.

In general, while receptor conversion can occur because of true molecular evolutionary changes associated with tumor progression, it can also be due to the inherent subjective nature of immunohistochemical evaluation. This can especially be witnessed when pathologist-assigned scores are close to the cut-off values of calling a tumor positive or negative for any given marker and was the reason for the perceived discrepant AR status in 2/5 cases in this study. This is a well-known and unresolved problem in the evaluation of biomarkers by pathologists where intra- and inter-observer variability can lead to discrepant results. This has been especially true for the assessment of the proliferation fraction (MIB-1) in BC [20, 21].

Other issues leading to potential discordant scores between matched samples is pre-analytical handling of the specimens and whether the specimens were optimally fixed, which can impair staining [22]. Tumor heterogeneity also plays an important role in interpreting results obtained from few selected regions in a tumor. Previous studies have demonstrated that 4 to 5 tumor punches on an array was sufficient to validate the use of a biomarker on a TMA [23]. Therefore, our ngTMA harbored multiple tissue punches to represent the tumor. However, the use of ngTMA to validate protein expression bears advantages and disadvantage.

Lastly, since we observed that 3/17 (17.6 %) AR negative primary BCs showed an AR positive lymph node metastases, we recommend re-testing of previous AR negative tumors. Additionally, re-testing of BCs with morphologically mixed components would be desirable. It has to be discussed with the oncologists if AR positive primary tumors need to be re-assessed on metastases or recurrences.

## Conclusions

In summary, AR positivity is very frequently found in BC regardless of the disease site and immunophenotype. Therefore, AR targeted therapy might be a warranted treatment option for many BC patients. Furthermore, it appears that AR status is highly preserved during tumor progression but discordance between primary and metastatic sites may occur in a small fraction of tumors. Hence, we recommend re-testing of AR status in previously AR negative primary tumors and BCs with morphologically mixed components.
